# Supplementary material for: DNA translocation mechanism of the MCM complex and implications for replication initiation
Source: Nat Commun. 2019 Jul 15;10:3117. doi: 10.1038/s41467-019-11074-3 (PMC6629641; doi:10.1038/s41467-019-11074-3)
Supplement: Supplementary file 1 — Supplementary Information [file 41467_2019_11074_MOESM1_ESM.pdf]

DNA translocation mechanism of the MCM complex and implications for replication initiation

Supplementary information

Martin Meagher[1†], Leslie B. Epling[1,2†], Eric J. Enemark[1*]

[1]Department of Structural Biology, St Jude Children's Research Hospital, 262 Danny Thomas Place, Mail Stop 311, Memphis, TN 38105, USA

[2]Present address: Incyte Research Institute, 1801 Augustine Cut-off, Wilmington, DE 19803, USA

†These authors contributed equally to this work.

*To whom correspondence should be addressed: eric.enemark@stjude.org

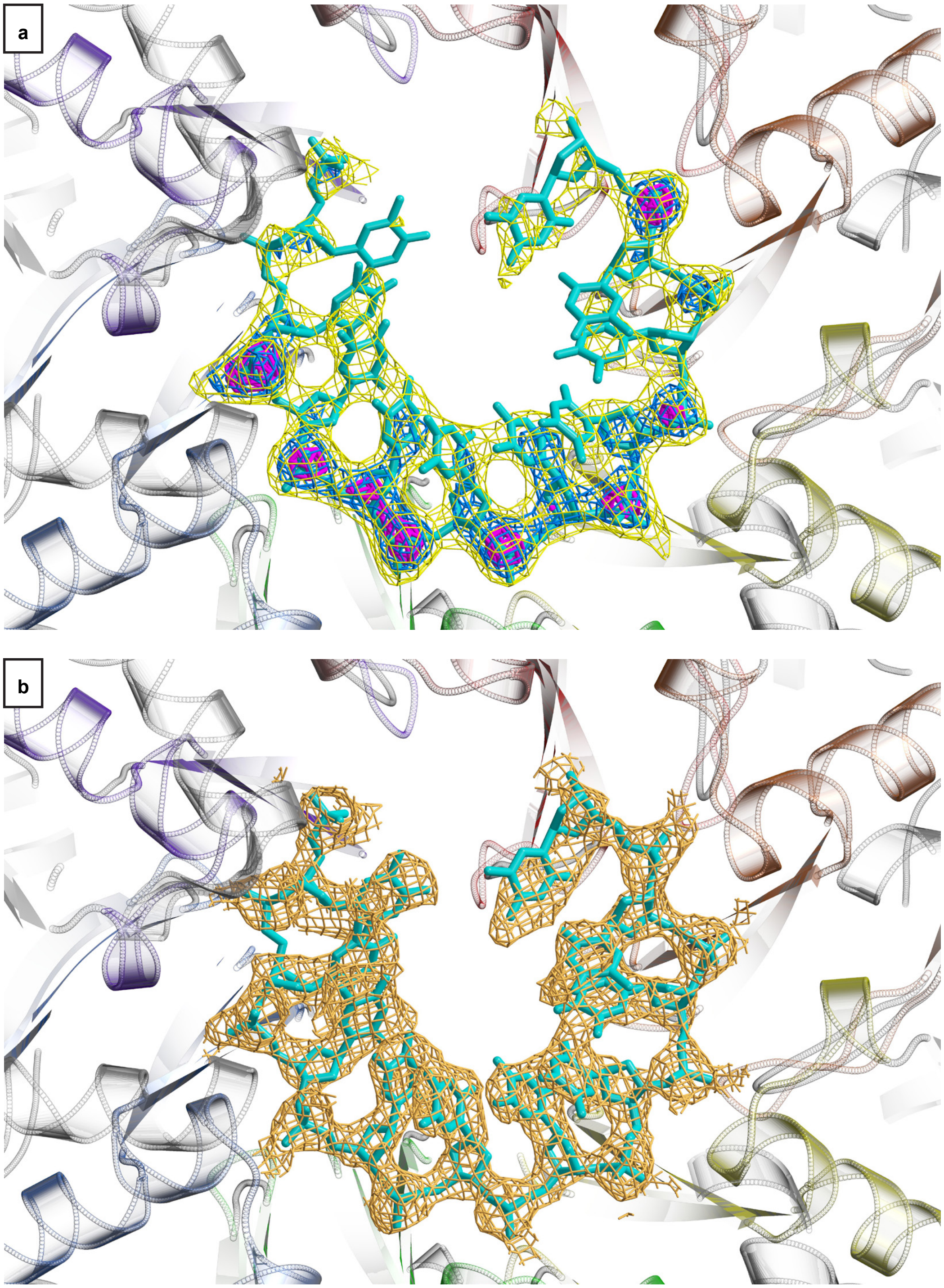

Supplementary Figure 1. Clear ssDNA electron density in the MCM central channel.

a, Fo-Fc difference electron density prior to inclusion of the ssDNA in the model contoured at 3-sigma (yellow), at 5-sigma (blue), and 7-sigma (magenta). All 10 phosphates are visible at 5-sigma with 7 visible at 7-sigma. **b**, Feature-enhanced electron density map[1] based on the final model. The maps are drawn around the final model with the ssDNA in stick and the protein in transparent cartoon. Both figure panels were generated with Bobscript[2] and rendered with Raster3D[3].

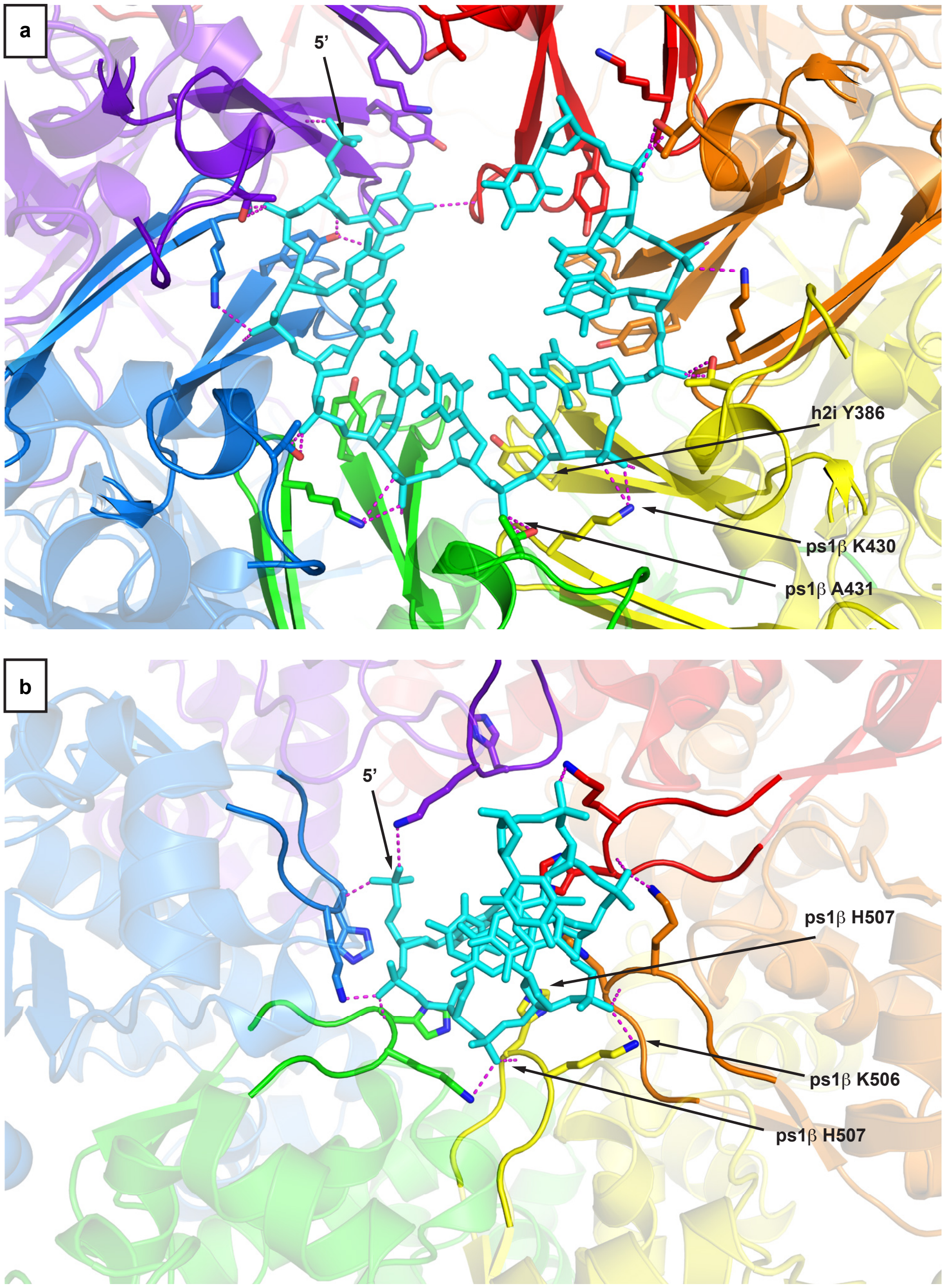

Supplementary Figure 2. Comparison of ssDNA binding by MCM and papillomavirus E1.

a, The MCM AAA+ domain uses 2 hairpins (h2i and ps1β) to bind ssDNA with an increment of 2 nucleotides per subunit. **b**, The E1 AAA+ domain binds ssDNA with its ps1β hairpin in a 1 nucleotide per subunit increment (PDB: 2GXA[4]). Both helicases use an equivalent binding mode between the ps1β and ssDNA, consisting of a main-chain amide binding one ssDNA phosphate (MCM A431 and E1 H507) and a lysine side-chain binding the phosphate of the next ssDNA nucleotide (MCM K430 and E1 K506). An aromatic residue of the MCM h2i (Y386) with intriguing partial conservation (see Supplementary Fig. 3) stacks on the ssDNA sugars similar to the side-chain of E1 H507. Both figure panels were prepared with PyMol[5].

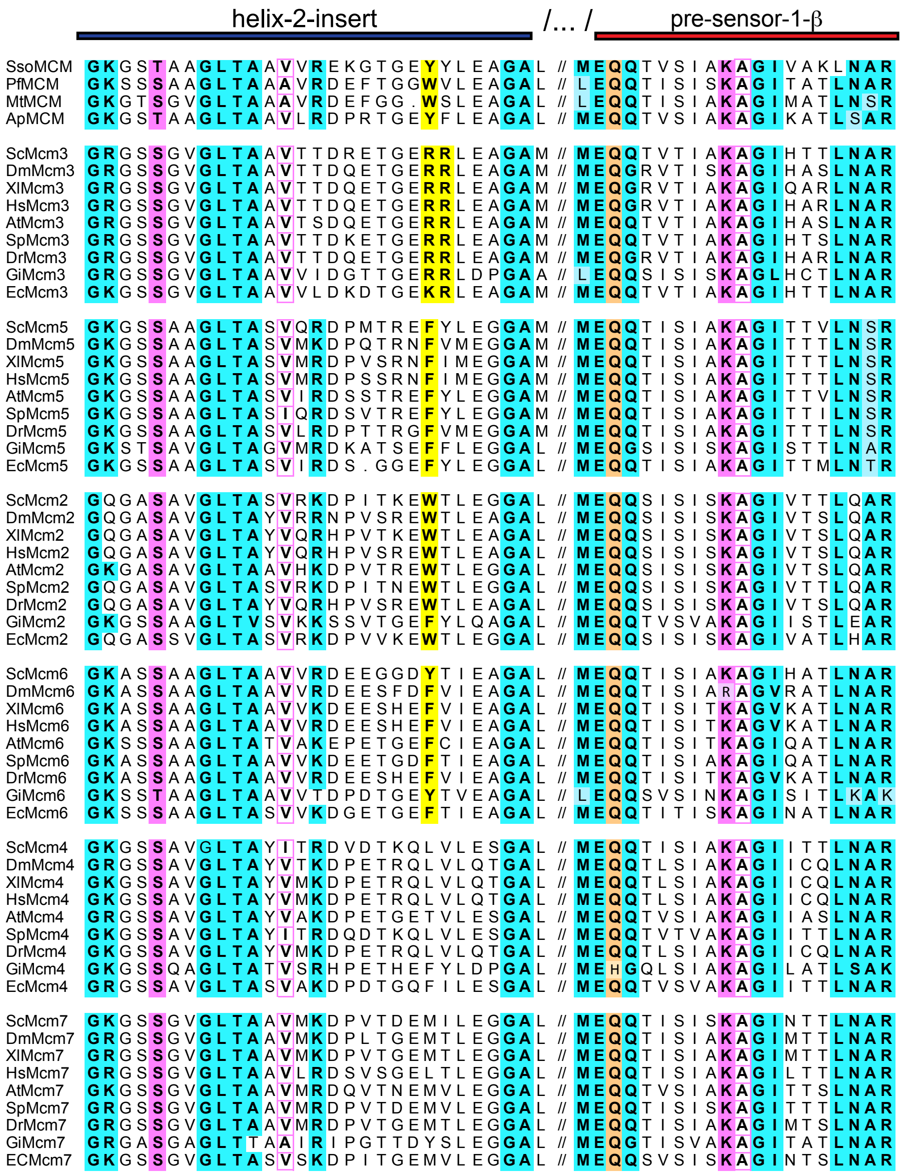

Supplementary Figure 3. The ssDNA-binding atoms are conserved in all MCM complex subunits.

The hydroxyl side chain (T369) and main-chain amide (V377) of the h2i, and the amino side-chain (K430) and main-chain amide (A431) of the ps1β are conserved in all MCM complexes (conserved side-chains are shaded magenta and conserved main-chain amides- non-proline- are boxed magenta). A conserved glutamine of the ATPase site (Q423) is shaded orange. Archaeal sequences are followed by eukaryotic MCM families in predicted descending staircase order immediately upon Cdc45 and GINS recruitment (see text analysis and Fig. 8): Mcm3, Mcm5, Mcm2, Mcm6, Mcm4, Mcm7. In this order, the aromatic residue on the tip of the h2i (Y386) is conserved as double-arginine in Mcm3 at the top of the staircase, followed by conserved aromatics at the next three staircase positions in Mcm5, 2, and 6 (yellow). Sso=*Sulfolobus solfataricus*; Pf=*Pyrococcus furiosus*; Mt=*Methanothermobacter thermautotrophicus*; Ap=*Aeropyrum pernix*; Sc=*Saccharomyces cerevisiae*; Dm=*Drosophila melanogaster*; Xl=*Xenopus laevis*; Hs=*Homo sapiens*; At=*Arabidopsis thaliana*; Sp=*Schizosaccharomyces pombe*; Dr=*Danio rerio*; Gi=*Giardia lamblia*; Ec=*Encephalitozoon cuniculi*.

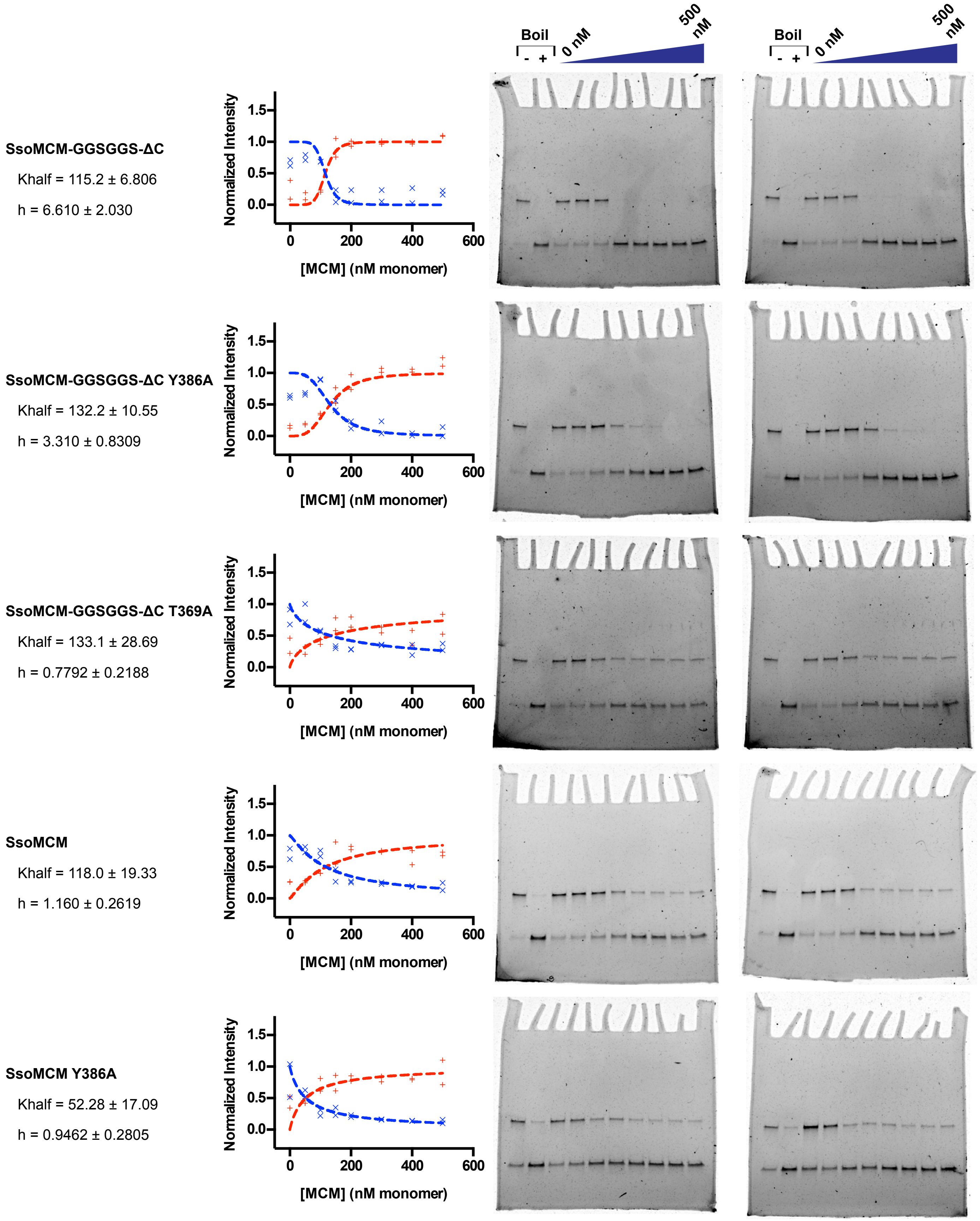

Supplementary Figure 4. See following page for title and legend.

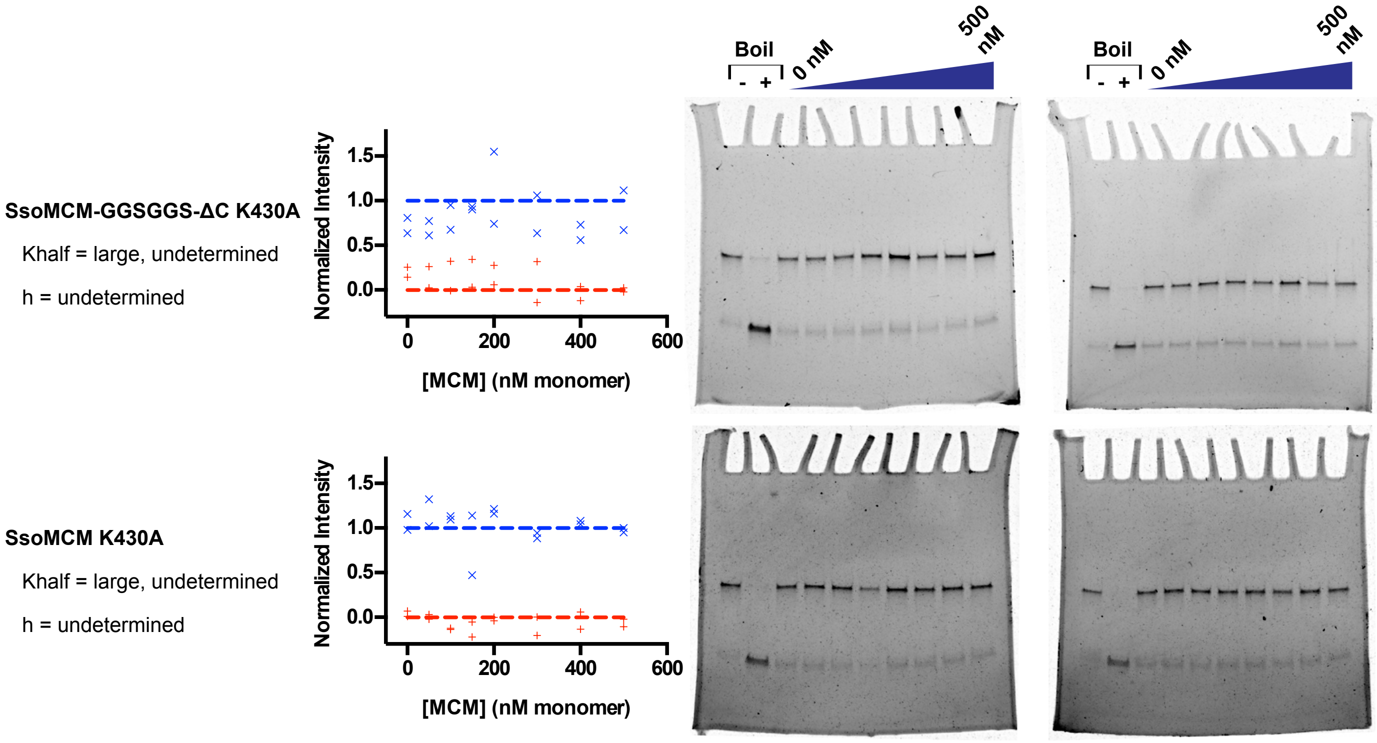

Supplementary Figure 4. Strand displacement assays for the proteins and mutants.

The strand displacement activity of each protein with a Y-shape substrate was measured by equivalent titration of each protein sample. Each assay was performed in duplicate to generate two gel images. The intensities of the upper band (intact Y-shape substrate) and lower band (displaced strand) were integrated, normalized, and fit to two simultaneous Hill equations to provide two parameters for each protein (K_{half} and h) and an 8:1 data/parameter ratio:

Bottom band (red): $[MCM]^h(K_{half}{}^h + [MCM]^h)^{-1}$; Top band (blue): $1 - [MCM]^h(K_{half}{}^h + [MCM]^h)^{-1}$, with [MCM] as the monomeric MCM concentration.

The monomeric [MCM] concentrations of lanes 3-10 are 0, 50, 100, 150, 200, 300 ,400, and 500 nM, respectively. Reactions consisted of 25 mM HEPES (pH = 7.6), 10 mM NaCl, 5 mM $Mg(OAc)_2$, 4 mM ATP, and 3.7 nM fluorescein-labeled DNA substrate and were incubated at 69 °C for 60 minutes. The normalized intensities of both replicates are plotted for each protein with top band intensities as blue X and bottom band intensities as red plus. The fit (GraphPad Prism) for each species are shown in dashed line.

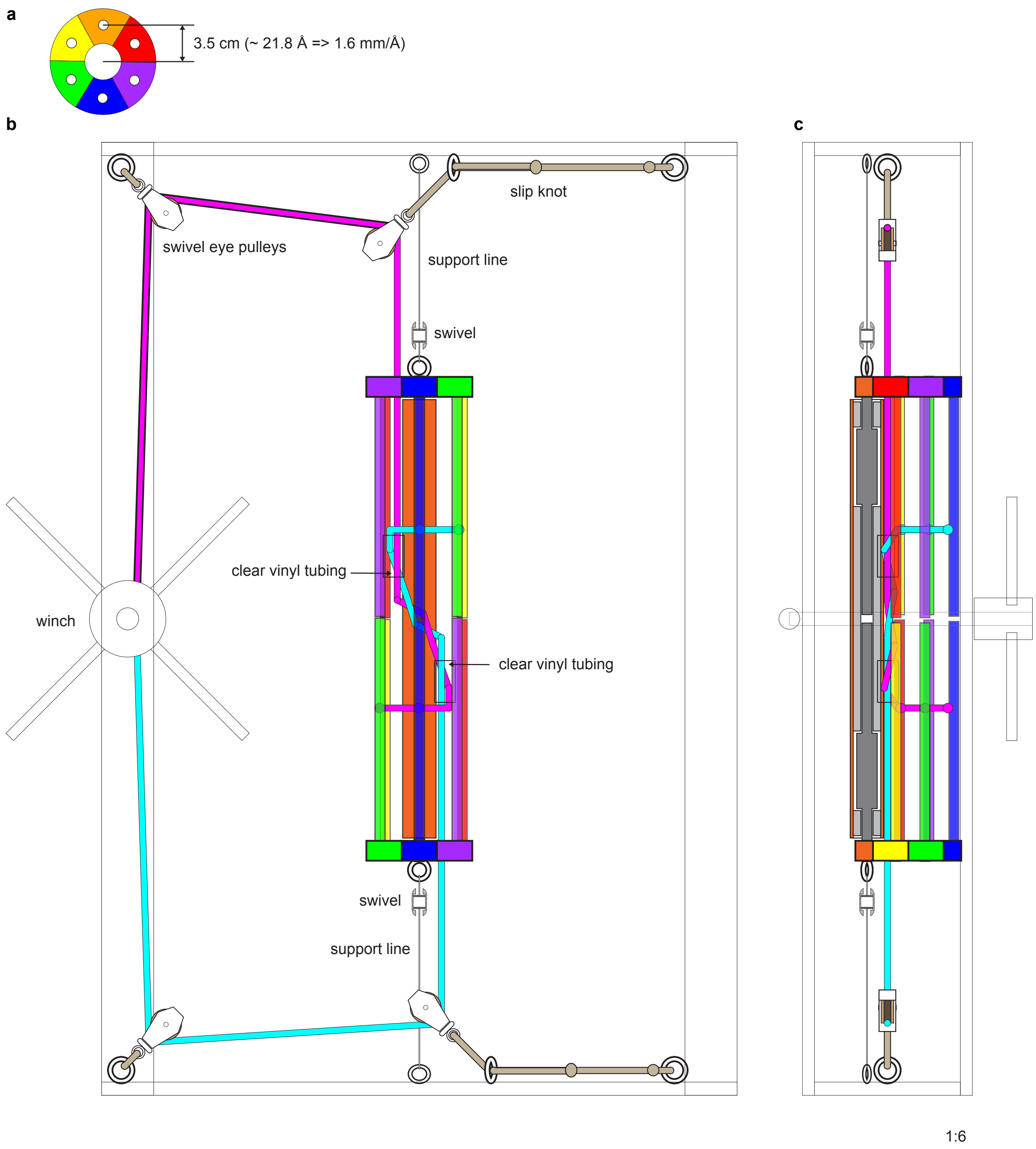

Supplementary Figure 5. Scale diagrams (1:6 scale) of the physical model of Supplementary Movie 6

a, Each hexamer base has a central hole for a strand to pass through and 6 peripheral holes for anchoring rods. The peripheral hole distances roughly correspond to the Zn atom distances and set the overall scale of the model. **b**, The model has cyan and magenta strands attached to the MSSB of the blue, green, and yellow rods of one hexamer and also to a winch that provides translocation-based tension. **c**, Perpendicular view including a cutaway view of the hinge.

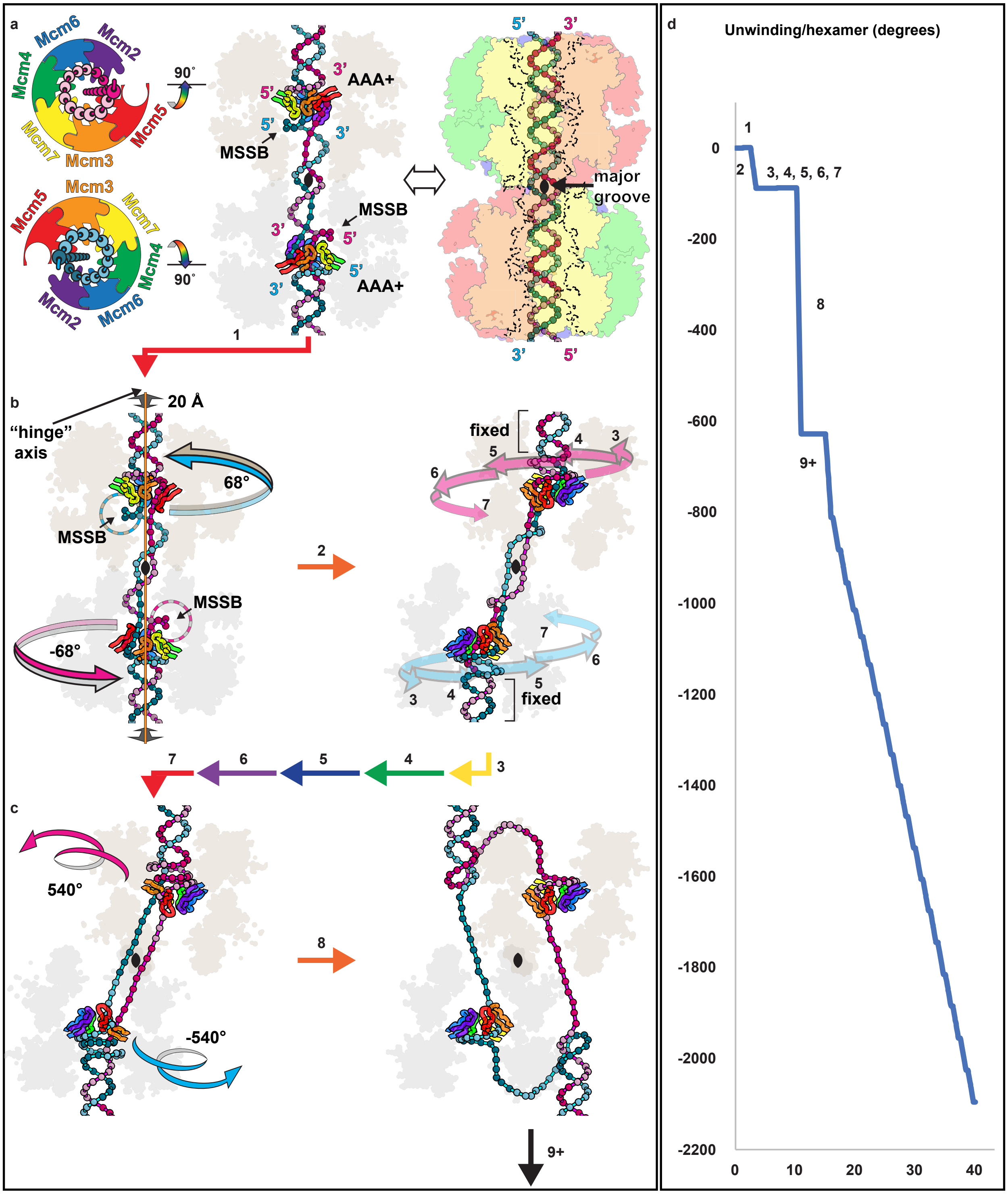

Supplementary Figure 6. Calculated degrees of unwinding associated with each step of the proposed initiation mechanism

a-c, Stepwise transformation of a double-hexamer encircling dsDNA to single hexamers that encircle ssDNA, as described in Fig. 8. **d**, The cumulative unwinding is plotted for each step, illustrating two discrete steps of significant unwinding, steps 2 and 8.

Supplementary Methods

This section provides specialized details for the generation of the Supplementary Movies to allow interested readers to reproduce the videos from the PDB coordinate file. In particular, the basis for the coordinate transforms to illustrate the fundamental steps are provided.

Supplementary Movie 1 generation

Stereoimages of the final model were rendered for each ATPase with each view calculated by least-squares superposition of the Walker-A/B and sensor-1 elements of the site. The images included the electron density of a Feature Enhanced Map (FEM)[1] calculated based on the final refinement colored according to subunit or nucleotide proximity. The six stereoimage pairs were converted to Supplementary Movie 1 by Adobe Premiere Pro.

Supplementary Movie 4 generation

Coordinate files of the six distinct states of the rotary translocation mechanism (Fig. 4) were generated. First, the crystal structure coordinates were rotated and translated to place the channel axis along the Z-axis. Specifically, the axis that permutes the six OB-folds (residues 120-133 and 191-265) was placed coincident with the Z-axis by the programs LSQMAN[6] and MOLEMAN[7] in a procedure described previously[8]. The crystallographically observed DNA model was expanded by adding additional nucleotides of ssDNA to each end such that the terminal positions sat on the Z-axis.

A segment of idealized B-form DNA was generated in Coot[9]. For one strand, the translation vector defined by the last two phosphates of the 3'-end was used to place additional nucleotides at the 3'-end. The other strand was extended at the 5'-end by adding nucleotides that were rotated and translated to maintain consistent phosphate-phosphate distances. The extent of rotation smoothly decayed from 36° by scaling each successive nucleotide rotation by 0.8125. Collectively, the fork DNA unit was translated to place the terminal dsDNA phosphate of the first strand on the origin of the coordinate system. It was then rotated about the Z-axis to place the 3'-phosphate of the first strand on the YZ-plane. Lastly, it was rotated about the X-axis to place the 3'-phosphate on the Z-axis.

The protein:ssDNA model, with its channel aligned to the Z-axis, was translated along the Z-axis to place the 5'-end of the ssDNA at a position that allowed good bonding geometry with the 3'-end of the fork DNA model (see above). The protein:ssDNA model was then rotated about the Z-axis to place the interface between chains E and F along the 5'-arm of the fork model. The full DNA model was then regularized to good geometry with Coot[9] while maintaining fixed positions for the crystallograpically observed DNA portion.

The following procedure was applied to the protein:ssDNA portion of model in five iterations to generate the other coordinate files comprising the six states. The operator that provided explicit least-squares permution (B->A; C->B; D->C; E->D; F->E; and A->F) of the OB-folds was applied to the protein model and also to the portion of the translocating ssDNA strand not within the fork DNA model (see above). The protein coordinate chain names were updated (B->A; C->B; D->C; E->D; F->E; and A->F) to maintain a consistent chain name at each subunit location. The coordinate positions of the other DNA nucleotides were left unchanged. All the DNA nucleotide numbers were updated by subtracting 2 from each position on the leading strand and adding 2 to each position on the lagging strand. The overall procedure provides the appropriate

transformation to generate the next sequential state, with each hairpin descending one increment of the staircase; the bottom hairpin moving to the top; translocation of 2 nucleotides of ssDNA through the channel (leading strand); addition of 2 nucleotides of ssDNA to the excluded strand (lagging strand); and a net unwinding of 2 base-pairs of dsDNA. One step of the process is illustrated in Fig. 6. To make the movie appear more smooth, intermediate coordinate files were interpolated. Most aspects were by simple cartesian interpolation. The dsDNA interpolation was by incremental rotation about the DNA helical axis and incremental translation along this axis (for example, 3.6° rotation and 0.338 Å translation if dividing one idealized B-DNA base step over 10 increments). At the ATPase sites, each transition provides one ATP hydrolysis, one ADP release, and one ATP-binding event. To illustrate that these three ATPase site events could occur in a non-concerted fashion, which we consider more plausible than three concerted molecular events, a pause was included between each step in the cycle. This pause emulates the timing suggested in the analysis of E1[4] where two crystallographically different hexamers show 12 separate states that oscillate between actions of ATP-binding and ATP-hydrolysis. Images of the coordinate files were generated by PyMOL[5] and then converted to Supplementary Movie 4 by Adobe Premiere Pro.

Supplementary Movie 6 generation

Schematic diagrams of the model are presented in Supplementary Fig. 5. Two identical hexamer models were constructed from readily available wood and piping materials. Seven holes were drilled in a wooden circle: a central 1-3/8-inch diameter hole and six 3/8-inch diameter holes around the periphery. Oak dowels, 3/8-inch diameter X 9-inches long, were glued to the 3/8-inch drilled holes and anchored with a lateral screw. One dowel of each model was used to create a hinge joint with PEX tubing, PVC pipe, and adhesive. The components of the resulting double-hexamer model were colored according to the double-hexamer of Fig. 8. The top and bottom of the hinge axis were attached to vertically plumb positions of a wooden frame with fishing line, screw eyes, and barrel swivels to ensure torsional isolation of the model from the frame. Magenta and light blue chute cords were used to represent the DNA strands. One end was threaded through a vinyl tube at the position of the red subunit to illustrate the action of the AAA+ domain on this strand (see below). The other end was tethered to the blue, green, and yellow subunits to illustrate binding to the MSSB. The model is consistent with the starting configuration of Fig. 8 in linking number topology and in strand polarities. The translocation strand of each hexamer was threaded vertically through the central hole of the ring and then through sequential swivel eye pulleys and finally attached to a rod as a winch to pull both strands simultaneously with equivalent tension. A video showing the action of the model was recorded and assembled by the St. Jude Department of Biomedical Communications.

Supplementary Movie 7 generation

The pre-activation species consists of an MCM double-hexamer encircling dsDNA, and our crystal structure provides a view of the post-activation species with the MCM helicase engaged to ssDNA in the form expected for a DNA-unwinding complex. We sought to elucidate the most elementary transformation between these two conditions based on four basic criteria. First, the two hexamers act on the encircled DNA and with each other in fundamentally the same way. More specifically, a double-hexamer dyad and the DNA dyad coincide, and this dyad is essentially retained during the transformation. Second, the retained dyad axis for the archaeal double-hexamer belongs to the same class as the dyad of the eukaryotic Mcm2-7 double-hexamer (details below). Third, the constituent DNA strands bind to the MCM AAA+ domain hairpins as shown in this manuscript and also bind to the MCM N-terminal domain MSSB with a

topology and polarity illustrated previously[10] (Fig. 7). Fourth, the process begins with a configuration that emulates positioning Mcm5 at the top of the AAA+ domain staircase, which is expected immediately upon ATP binding at the Mcm5/2 ATPase site[11], while the MSSB-DNA interactions occur at subunit positions that emulate Mcm6, Mcm4, and Mcm7[10].

Archaeal MCM double-hexamers consist of 12 identical subunits arranged with D_6-symmetry with a single 6-fold axis through both hexamers and 6 perpendicular 2-fold axes between the hexamers. These perpendicular twofold axes fall into 2 classes (C2' and C2'';[12]) with three twofold axes passing through the hexamer:hexamer interfaces, and three twofold axes passing between the interfaces and bisecting the first 3 twofold axes. Upon encircling DNA, the complex is no longer 6-fold symmetric because the DNA is not 6-fold symmetric. Consequently, only a single twofold symmetry axis is allowed for a double-hexamer when DNA is encircled. No prior structural data for archaeal MCMs indicate which class of twofold axis would persist in an archaeal MCM double-hexamer bound to DNA-- specifically whether such a twofold passes through two interhexamer interfaces or sits in the middle of these positions (C2' or C2''). In contrast, eukaryotic MCM double-hexamers consistently and clearly indicate that the double-hexamer dyad axis passes through two hexamer interfaces: Mcm3:Mcm3' and Mcm6:Mcm6'[13-15]. Based on this property, we infer that the lone twofold axis of the archaeal MCM double hexamer encircling dsDNA belongs to the same class and bisects two subunit interfaces. In Fig. 8, this dyad passes through the inter-hexamer interfaces of the two orange subunits and the two blue subunits.

Using this condition, a double hexamer molecular model was constructed by placing two copies of our MCM:ssDNA crystal structure in a 2-fold symmetric double hexamer based on the interface of the two hexamers in the crystal structure of the N-terminal domain double-hexamer of *Pf*MCM (PDB 5IY0[16]) as depicted in Fig. 8. The *Pf*MCM double-hexamer structure was positioned with its centroid on the coordinate system origin and a dyad passing through two of the interhexamer interfaces coincident with the X-axis. Two copies of the *Sso*MCM-GGSGGS-ΔC:ssDNA structure were positioned by the least-squares superposition of the 6 OB-folds onto the 6 OB-folds of each *Pf*MCM hexamer. For this placement, the orientation about the OB-fold permution axis was chosen to place the highest hairpin of the staircase one subunit clockwise, as viewed from the N-terminal side of the hexamer, to the subunit at the interhexamer dyad to emulate positioning of Mcm5 at the top. The placement of Mcm5 hairpins at the top of the staircase is expected immediately upon ATP binding at the Mcm5/Mcm2 ATPase site. The basis for this expectation is readily seen in Fig. 4 where the transition of an ATPase site from empty (E) to ATP-bound (T) drives the associated hairpins to the top of the staircase.

The resulting double-hexamer was translated along the X-axis to place the centroids of the OB-folds of each constituent hexamer in the YZ-plane, and the model was then rotated about the X-axis to place the YZ-plane projection of the OB-fold permution axis parallel to the Z-axis. This last rotation about the X-axis was calculated by determining the direction of the polar rotation axis with LSQMAN[6] and applying the necessary rotations with MOLEMAN[7]. Although the channel axes of the two hexamers of the resulting double-hexamer model appear co-axial, this reflects the approximate collinearity of the original N-terminal domain double hexamer model used for this construction, and is not inherent in the procedure, as observed in the model for Supplementary Movie 8 (see below).

An idealized B-form double-stranded DNA model was generated with Coot[9], which automatically placed the DNA helical axis along the Z-axis. In order to make the DNA dyad axis coincide with that of the double-hexamer model, the DNA molecule was translated to place its centroid at the

coordinate system origin and was rotated about the Z-axis to align its dyad axis with the X-axis. This alignment can be achieved with the major groove or the minor groove facing outward. Both versions were constructed and inspected for compatibility with how DNA binds to the AAA+ domain in this report, and how DNA binds to the MSSB of the N-terminal domain described previously[10], and the inferred starting staircase arrangement (see above). The version with the major groove facing outward provided the desired arrangement and topology and was selected (see Fig. 8).

The cumulative number of degrees of unwinding per hexamer was calculated as the cumulative rotation of the dsDNA ahead of one hexamer about the dsDNA helical axis. Specifically, a fixed range of dsDNA nucleotides in the model were translated to place the centroid at the origin of the coordinate system. The tangent of the angle defined by the X and Y coordinates of a DNA phosphate defined the absolute rotation angle of the current model about the dsDNA helical axis. The difference of this angle from that of the previous model in the series was added to a running cumulative number of degrees of unwinding. Results of such analysis are illustrated in Supplementary Fig. 6 and Supplementary Movie 8.

The transformation of Supplementary Movie 7 proceeds through three fundamental stages that are defined by the overall degree of dsDNA unwinding. The first stage has no net dsDNA unwinding and consists of an MCM double-hexamer encircling dsDNA in a species that first engages binding to the DNA strands with one strand bound to the AAA+ domain in the staircased arrangement of our present crystal structure and places the other strand at the MSSB of the N-terminal domain as shown in our earlier crystal structure[10]. Although base-pairs must be separated to some extent to achieve this DNA arrangement, the DNA molecule is topologically equivalent to B-form dsDNA, and no net unwinding has occurred because the dsDNA portion has not rotated about its helical axis (see above and step 1 of Fig. 8 and Supplementary Fig. 6).

In order for each hexamer to pull its translocation strand from the central channel of the opposing hexamer through consistent interfaces, two consistent interfaces must align to face each other to serve as the strand exit interfaces. Six basic permutations are possible to achieve this alignment (Supplementary Movie 5). Two involve a hinge rotation that slightly unwinds the DNA; two perform a slide with no associated change in DNA winding; and two perform a hinge rotation that winds the DNA further. We consider the hinge rotation in the direction of unwinding as the most likely candidate because it would provide the first of two unwinding steps towards activation[17], and also because it leads to a capture of the two strands at a specific subunit interface adjacent to the hinge to precisely ensure the correct interface. This hinge rotation is about the inter-hexamer interface that sits on the X-axis dyad and is based on inspection of physical models, including that of Supplementary Movie 6 and Supplementary Fig. 5. The physical model (Supplementary Movie 6) demonstrates that a hinge rotation about the orange interhexamer interface captures the strands at the orange and yellow subunit interfaces of each hexamer.

For the models of Supplementary Movie 7, the hinge rotation was generated by rotation of one hexamer about an axis parallel to the Z-axis and passing through the centroid of the two zinc atoms of the two subunits of the double hexamer (orange subunit in Fig. 8 and Supplementary Movies 7, and 8). The rotation was by an amount that brought the immediately clockwise (as viewed from the C-terminal side) intersubunit interface approximately along the YZ-plane (see orange and yellow hairpins in step 2 of Fig. 8). Coordinates for the second hexamer were obtained by applying the X-axis dyad. Because the dsDNA outside the hexamer rotates about its helical axis along with the protein complex during this hinge rotation, an unwinding equivalent to the degrees of hinge rotation is calculated for the degrees of unwinding (see above).

During each sequential step of this stage, 2 nucleotides of ssDNA are translocated from above the staircase to below the staircase. This was achieved by modifying the procedure employed for Supplementary Movie 4. The nucleotides were renumbered by adding 2 to each nucleotide of the lagging strand and subtracting 2 from the leading strand. In addition, 2 nucleotides were deleted above the staircase, and 2 nucleotides were added below the staircase. The second hexamer and second half of the DNA molecule were obtained by the X-axis dyad, and the resulting DNA molecule was regularized to good geometry in Coot[9] while the crystallographically observed DNA nucleotides were held fixed. During these steps, the leading strand wraps around the lagging strand below the staircase, but no net unwinding of the dsDNA is generated (steps 3-8, Supplementary Fig. 6) because the dsDNA does not rotate about its helical axis during these steps.

During these steps, the leading strand of each hexamer is successively pulled through the orange and yellow subunit interface of the opposing hexamer. Step 8 is special for this specific interface in that the ATPase site is placed into the empty (E) configuration, the stage when it is in its most open conformation (see Fig. 4). We suggest that the site must be in the empty state of the ATPase cycle at the time the lagging strand exits the interface. In addition, our molecular modeling indicates that the DNA located between the two hexamers runs out of slack precisely when this site is in this state. We propose that the DNA that has been coiled around the lagging strand in steps 2-8 unsprings all at once to provide the requisite strand escape needed to establish a strand-excluded species. To illustrate this transformation, the DNA was rotated about its helical axis and translated in the YZ-plane until it was outside of the hexamer. This step provides the largest number of degrees of DNA unwinding in the activation process because the dsDNA portion rotates approximately 1.5 turns about its helical axis. We note that the opening for the ATPase site empty state of our crystal structure is not expected to be large enough to permit passage of a dsDNA molecule, and hence this interface would need to open further than we have observed in our present crystal structure. Nevertheless, we expect that such a transformation would need to occur when the ATPase site is in the E state, and hence Supplementary Movie 7 provides an appropriate timing and our best framework for how the molecular transformation would occur.

Following the strand escape step of the movie, the two hexamers proceed independently in a fashion illustrated in Fig. 6 and Supplementary Movie 4. The net unwinding at each step (steps 9 onward) is approximately 72° per hexamer (the helical twist of 2 nucleotides of B-DNA), but this was modified slightly to smoothly bring the hexamer to an orientation where the lagging strand sits on top of the interface of the red and orange subunits. For this stage, coordinates of the ssDNA portions between the hexamers were generated to have approximately equally spaced nucleotides and a vertical step that followed a decay function. Nucleotides were tilted by an angle given by the inverse tangent of the vertical and horizontal distances to the preceding nucleotide. This procedure provided ssDNA that smoothly varied from a horizontal projection as the lagging strand of one hexamer to vertical projection as the leading strand of the other hexamer. The parameters for the mathematical functions were re-calculated at each step to account for the specific length of ssDNA and the specific hexamer-hexamer spacing.

Images of the successive coordinate files were generated (with the Z-axis vertical and the Y-axis horizontal) by PyMOL[5] as PNG format or Bobscript[2] as PS format. The Bobscript images were processed by Adobe Illustrator and then converted by Adobe Photoshop to PNG format. All images were then converted to a movie by Adobe Premiere Pro.

Supplementary Movie 8 generation

Supplementary Movie 8 starts from the established pre-activation structure of the Mcm2-7 double-hexamer encircling dsDNA as determined by cryo-EM (PDB 5BK4[15]). In this species, the AAA+ domain ps1β hairpins bind to the lagging strand, which needs to switch to the leading strand to generate the translocation species of Fig. 2. A model for the latter form of DNA-engagement was constructed based on the models of Supplementary Movie 7 and the cryo-EM structure of the *Sc*Mcm2-7 double-hexamer (PDB 3JA8[14]). The coordinates of a *Sc*Mcm2-7 double hexamer EM structure[14] was positioned with its dyad along the X-axis. Although each subunit of *Sc*Mcm2-7 differs, the OB-folds are highly conserved in structure, and the axis to permute these structural units was taken as the channel axis, analogous to the procedure of Supplementary Movie 7. The axis was determined by first aligning a copy of the *Sso*MCM OB-fold to each OB-fold of the *Sc*Mcm2-7 double-hexamer by LSQMAN[6]. The resulting model with identical residue construction provided a straightforward calculation of the axis of permution and assured that each subunit was weighted equivalently towards calculation the OB-fold centroid for this procedure. The calculated angle between the permution axis and the Z-axis was then applied to the double-hexamer model as an X-axis rotation to align its channels parallel to the Z-axis.

To obtain a model for the associated encircled dsDNA, the aligned *Sso*MCM OB-folds were used to elucidate the transformation for one hexamer of the archaeal MCM double-hexamer:B-DNA model (from step 1 of Supplementary Movie 7) such that the red subunit was matched to Mcm5. A second hexamer:B-DNA model was added based on the X-axis dyad. The central DNA between the two OB-folds was rotated by an angle defined by the tangent of the Y- and Z-coordinates of the centroid of the OB-folds. The geometry of the full dsDNA was regularized with Coot[9]. The resulting DNA is zigzag shaped with the exterior arms projected up and down, approximately parallel to the Z-axis and the middle segment at an angle intermediate between horizontal and vertical. This model directs the dsDNA major groove towards the Mcm3:Mcm3' interface.

To identify the minimal transformation between the species where the ps1β hairpins bind to the lagging strand to the one where they bind the leading strand, the RMSD for explicit alignment of the phosphorous atoms of the dsDNA molecules was calculated for all sequential permutations, and the best scoring alignment was selected. This coordinate model provides a unique MCM model for the movies because it does not adhere to a dyad axis on the X-axis. Instead, the dyad of the double-hexamer protein model slides and, to a lesser extent, rotates until it is coincident with the X-axis, and the dsDNA major groove faces the Mcm3:Mcm3' interface. Dyads of the protein model and the DNA are collinear at the start of the slide, with a DNA <u>minor groove</u> dyad facing the same direction as the Mcm3:Mcm3' interface, and the dyads are again collinear at the end of the slide, with a DNA <u>major groove</u> dyad facing the same direction as the Mcm3:Mcm3' interface, but the dyads are not collinear during the slide. This is the only step in Supplementary Movies 7 and 8 when a dyad relating the ring hexamers and a dyad of the DNA are not collinear during the transformation.

Analogous to the six states described above for the *Sso*MCM hexamer, six sequential states were constructed for Mcm2-7. These were generated by least-squares superposition of each of the six *Sso*MCM states onto the *Sc*Mcm2-7 double-hexamer[14] based on the OB-folds. In each case, the resulting positions of the six *Sso*MCM AAA+ domains were then used as the basis for least-squares superposition of the matched *Sc*Mcm2-7 AAA+ domain structure[14]. This procedure

produced 6 different molecular state models of *Sc*Mcm2-7 with a static N-terminal tier identical to the structure shown in the *Sc*Mcm2-7 double-hexamer[14] and AAA+ positions that follow the staircased positions of our crystal structure. The models are intended to illustrate molecular scale and that the process is overall physically reasonable. The Mcm2-7 models have not been subjected to minimization or modification following the simple docking. Nevertheless, the resulting models do not show significant clashes, and we note that the conservation among MCM AAA+ domains is very strong in both sequence and in structure. For models that include Cdc45 or GINS, Cdc45 and GINS subunits were positioned by aligning a CMG structure (PDB 3JC5[18]) to the above Mcm2-7 models based on the N-terminal tier. These models are also intended to illustrate molecular scale and to show the relative actions of the N-terminal tier, the AAA+ tier, and the DNA-- and that Cdc45 and GINS would not sterically conflict with any of the protein or DNA motions proposed here.

The events of Supplementary Movie 8 proceed largely according to Supplementary Movie 7. The hinge rotation at step 2 begins from the above double-hexamer with AAA+ domain staircase and proceeds to a model given by alignment of the matched staircase/ATPase state model of *Sc*Mcm2-7 (along with Cdc45 and GINS) onto the *Sso*MCM model of the conclusion of step 2 based on the N-terminal tier OB-fold positions. From this point forward, each *Sc*Mcm2-7 hexamer model was positioned similarly by alignment of the matched staircase/ATPase state model to a *Sso*MCM hexamer of Supplementary Movie 7 based on the OB-folds of the N-terminal tier.

The molecular models for Supplementary Movie 8 were obtained with MOLEMAN[7] and LSQMAN[6], and images were rendered (with the Z-axis vertical and the Y-axis horizontal) by Pymol. The cumulative degrees of unwinding were calculated for each model, and the result was added to a running plot that was converted to a PNG format graphic by the linux command convert. The graphic files were converted to a movie file by Adobe Premiere Pro.

Supplementary References

1 Afonine, P. V. *et al.* FEM: feature-enhanced map. *Acta Crystallogr D Biol Crystallogr* **71**, 646-666, doi:10.1107/S1399004714028132 (2015).

2 Esnouf, R. M. An extensively modified version of MolScript that includes greatly enhanced coloring capabilities. *J Mol Graph Model* **15**, 132-134, 112-133, doi:10.1016/S1093-3263(97)00021-1 (1997).

3 Merritt, E. A. & Bacon, D. J. Raster3D: photorealistic molecular graphics. *Methods Enzymol* **277**, 505-524 (1997).

4 Enemark, E. J. & Joshua-Tor, L. Mechanism of DNA translocation in a replicative hexameric helicase. *Nature* **442**, 270-275, doi:10.1038/nature04943 (2006).

5 The PyMOL Molecular Graphics System, Version 1.3r1 (2010).

6 Kleywegt, G. J. Use of non-crystallographic symmetry in protein structure refinement. *Acta Crystallogr D Biol Crystallogr* **52**, 842-857, doi:10.1107/S0907444995016477 (1996).

7 Kleywegt, G. J. Validation of protein models from Calpha coordinates alone. *J Mol Biol* **273**, 371-376, doi:10.1006/jmbi.1997.1309 (1997).

8 Miller, J. M., Arachea, B. T., Epling, L. B. & Enemark, E. J. Analysis of the crystal structure of an active MCM hexamer. *Elife* **3**, e03433, doi:10.7554/eLife.03433 (2014).

9 Emsley, P. & Cowtan, K. Coot: model-building tools for molecular graphics. *Acta Crystallogr D Biol Crystallogr* **60**, 2126-2132, doi:10.1107/S0907444904019158 (2004).

10 Froelich, C. A., Kang, S., Epling, L. B., Bell, S. P. & Enemark, E. J. A conserved MCM single-stranded DNA binding element is essential for replication initiation. *Elife* **3**, e01993 (2014).

11 Bochman, M. L. & Schwacha, A. Differences in the single-stranded DNA binding activities of MCM2-7 and MCM467: MCM2 and MCM5 define a slow ATP-dependent step. *J Biol Chem* **282**, 33795-33804, doi:10.1074/jbc.M703824200 (2007).

12 Cotton, F. A. *Chemical applications of group theory*. 3rd edn, (Wiley, 1990).

13 Sun, J. *et al.* Structural and mechanistic insights into Mcm2-7 double-hexamer assembly and function. *Genes Dev* **28**, 2291-2303, doi:10.1101/gad.242313.114 (2014).

14 Li, N. *et al.* Structure of the eukaryotic MCM complex at 3.8 A. *Nature* **524**, 186-191, doi:10.1038/nature14685 (2015).

15 Noguchi, Y. *et al.* Cryo-EM structure of Mcm2-7 double hexamer on DNA suggests a lagging-strand DNA extrusion model. *Proc Natl Acad Sci U S A* **114**, E9529-E9538, doi:10.1073/pnas.1712537114 (2017).

16 Meagher, M. & Enemark, E. J. Structure of a double hexamer of the Pyrococcus furiosus minichromosome maintenance protein N-terminal domain. *Acta Crystallogr F Struct Biol Commun* **72**, 545-551, doi:10.1107/S2053230X1600858X (2016).

17 Douglas, M. E., Ali, F. A., Costa, A. & Diffley, J. F. X. The mechanism of eukaryotic CMG helicase activation. *Nature* **555**, 265-268, doi:10.1038/nature25787 (2018).

18 Yuan, Z. *et al.* Structure of the eukaryotic replicative CMG helicase suggests a pumpjack motion for translocation. *Nat Struct Mol Biol* **23**, 217-224, doi:10.1038/nsmb.3170 (2016).

19 Abrahams, J. P., Leslie, A. G., Lutter, R. & Walker, J. E. Structure at 2.8 A resolution of F1-ATPase from bovine heart mitochondria. *Nature* **370**, 621-628, doi:10.1038/370621a0 (1994).

20 Kagawa, R., Montgomery, M. G., Braig, K., Leslie, A. G. & Walker, J. E. The structure of bovine F1-ATPase inhibited by ADP and beryllium fluoride. *EMBO J* **23**, 2734-2744, doi:10.1038/sj.emboj.7600293 (2004).

21 Stein, N. CHAINSAW: a program for mutating pdb files used as templates in molecular replacement. *Journal of Applied Crystallography* **41**, 641-643, doi:10.1107/S0021889808006985 (2008).

22 Afonine, P. V. *et al.* Towards automated crystallographic structure refinement with phenix.refine. *Acta Crystallogr D Biol Crystallogr* **68**, 352-367, doi:10.1107/S0907444912001308 (2012).
