# Supplementary material for: DNA translocation mechanism of the MCM complex and implications for replication initiation
Source: Nat Commun. 2019 Jul 15;10:3117. doi: 10.1038/s41467-019-11074-3 (PMC6629641; doi:10.1038/s41467-019-11074-3)
Supplement: Supplementary file 3 — Description of Additional Supplementary Files [file 41467_2019_11074_MOESM3_ESM.pdf]

Description of Additional Supplementary Files

File Name: Supplementary Movie 1
Description: Loop stereoview of the sequential rotary ATPase cycle of the MCM ring (0:06 loop; 530 KB; 600 x 300).

The subunit containing the Walker-A/B and sensor-1 residues is in yellow, and the adjacent subunit containing the arginine finger, sensor-2, and sensor-3 residues is in cyan. Nucleotide co-factors are in stick, and the magnesium ion is in magenta sphere. The electron density of a Feature Enhanced Map (FEM)[1] calculated based on the final refinement is shown colored according to subunit proximity. The density shows a clear binding site change mechanism analogous to that described for F1-ATPase[19,20]. The tight interfaces (T, T*) are defined by continuous density between the arginine finger (R473) and nucleotide. The loose interfaces (D) lack this continuous density but retain density between nucleotide and Q423. The empty interface (E) lacks both interactions.

File Name: Supplementary Movie 2
Description: Loop cartoon movie of 2 successive cycles of the ssDNA translocation mechanism (0:12 loop; 557 KB; 1080 X 1080).

The view is from the C-terminal side (beneath the staircase). The subunits are colored as in Fig. 2 with individual DNA nucleotides represented as cyan beads on a string. One bead is colored white to help illustrate ssDNA motion. Each subunit passes sequentially through a full ATP hydrolysis cycle (T -> T* -> D -> E -> T; see Fig. 3), moving its two DNA-binding hairpins to escort 2 nucleotides of ssDNA through the central channel. To illustrate that these three ATPase site events could occur in a non-concerted fashion, which we consider more plausible than three concerted molecular events, a pause was included between each step in the cycle. This pause emulates the timing suggested in the analysis of E1[4] where two crystallographically different hexamers show 12 separate states that oscillate between actions of ATP-binding and ATP-hydrolysis. The frame of reference is looking directly along the ring channel from the C-terminal side a fixed distance from the ring.

File Name: Supplementary Movie 3
Description: Illustration of the transformation of a helix-2-insert from a horseshoe to a helix conformation (0:02; 450 KB; 1280 X 1280).

The close proximity of the DNA-binding modules of adjacent subunits when binding ssDNA is compatible with the helix h2i conformation of our present structure, but not with the horseshoe conformation at several subunits of the Mcm2-7 double-hexamer. The movie illustrates the transition of the Mcm5 h2i horseshoe in the DNA-bound Mcm2-7 double-hexamer (PDB 5BK4[15]) to the helical conformation in the model of Fig. 5b constructed from our present structure with Chainsaw[21]). The Mcm5 h2i is in bright red with the rest of Mcm5 in light red. The Mcm2 ps1β is in bright purple with the rest of Mcm2 in light purple. During the transformation, the Mcm2 ps1β closely approaches the Mcm5 h2i and is no longer compatible with a horseshoe conformation. The final position provides parallel β-sheet interactions that serve as staircasing interactions[4] between the Mcm5 h2i and the Mcm2 ps1β. The movie was generated with a cartesian-based morph by LSQMAN[6], and the models were subjected to brief geometry minimization with

phenix.geometry_minimization[22]. Images were then prepared with Bobscript[2] and Raster3D[3] and converted to a movie by Adobe Premiere Pro.

File Name: Supplementary Movie 4
Description: Loop movie for the proposed mechanism for DNA unwinding by the MCM complex (0:06 loop; 7.2 MB; 1920 X 1080).

Two perpendicular perspectives are illustrated. Each subunit is shown in cartoon and uniquely colored as in Fig. 2 with the two DNA strands in cyan and magenta spheres. Every 12th DNA nucleotide is colored white to help show strand motion. The ring translocates the cyan strand downward to generate a pulling force that rotates the dsDNA about its helical axis (similar to a pulley wheel), which fundamentally unwinds the two strands. A pause is included between each step as described for Supplementary Movie 2. Both perspectives use a frame of reference that is at a fixed position relative to the centroid of the OB-folds. One is viewed perpendicular to the plane defined by the channel axis and the dsDNA helical axis, and the other is parallel to this plane. These reference frames are advantageous because they can illustrate an indefinite loop and also clearly show how the ring and DNA move with respect to each other. However, the ring likely would not sit at a fixed position when viewed from the absolute reference frame.

File Name: Supplementary Movie 5
Description: Six scenarios align equivalent interfaces of the MCM double-hexamer encircling DNA (0:00.86; 379 KB; 1020 x 1080).

Alignment of equivalent interfaces enables the DNA strands to be pulled through the same interface of each hexamer. Four twist scenarios proceed with a hinge that provides a topological barrier to capture the DNA strands at its interface with an adjacent subunit (see Supplementary Movie 6). Due to the DNA rotation, these hinge scenarios either unwind DNA or overwind DNA, depending on twist direction. Two slide scenarios provide zero net change to the DNA winding. Each panel illustrates a view down the dyad axis with the dsDNA major groove facing outward (top view of each panel) and a view down the channel axis (bottom view). **a**, Rotation in the DNA unwinding direction about a hinge at the major groove side of the DNA (orange) to align the yellow/orange interfaces. **b**, Rotation in the DNA unwinding direction about a hinge at the minor groove side of the DNA (blue) to align the purple/blue interfaces. **c**, An interhexamer slide with no change to DNA winding to align the red/purple interfaces. **d,** An interhexamer slide with no change to DNA winding to align the green/yellow interfaces. **e**, Rotation in the DNA winding direction about a hinge at the major groove side of the DNA (orange) to align the orange/red interfaces. **f**, Rotation in the DNA winding direction about hinge at the minor groove side of the DNA (blue) to align the blue/green interfaces. The analysis applies identically to eukaryotic Mcm2-7 due to the equivalent C_1-symmetry of the constituent hexamers when encircling DNA and also an equivalent dyad to relate the two hexamers. For Mcm2-7, the hinge scenarios would involve rotation about one of the symmetric interhexamer interfaces as a hinge (either Mcm3:Mcm3’ or Mcm6:Mcm6’). MCM subunits are uniquely colored in cylinder representation sized to the Zn-atom positions of a double-hexamer (PDB 5IY0[16]). The DNA strands are represented as cyan and magenta beads on a string according to the phosphorous atom positions of DNA molecules sized to the same scale as the double-hexamer model. Molecular images were generated by PyMOL[5].

File Name: Supplementary Movie 6
Description: Physical model demonstrating a facile hinge rotation in the double-hexamer upon application of tension to each strand (0:22; 4.4 MB; 1920 x 1080).

The color scheme is as in Fig. 2 and Fig. 8. Rotation of a winch pulls the cyan strand downward and the magenta strand upward to represent the action of the AAA+ tiers. A white stripe is included on each strand to assist visualizing the motion. These strand translocation directions assign the polarity of each strand due to the 3'->5' polarity of the MCM helicase. Each strand is tethered at three approximately coplanar positions on the opposing hexamer to provide a configuration and polarity that matches the previously reported ssDNA-binding by the N-terminal domain MSSB (clockwise 5' -> 3' when viewed from the side opposite the double-hexamer interface). When placed under tension, the strands pull on the opposing hexamer to rotate the two hexamers with respect to each other about a hinge. See Methods and Supplementary Fig. 5 for model details. The frame of reference is approximately down the colinear dyad axes of the two hexamers and the two DNA strands. The zoom-in view is at an angle to reduce obscuring of details.

File Name: Supplementary Movie 7
Description: Proposed MCM:DNA aspects of replication initiation (0:39; 18.4 MB; 1860 X 1080).

The color scheme is as in Fig. 2 and Fig. 8. The movie illustrates a stepwise transformation of a double-hexamer encircling dsDNA to single-hexamers that encircle ssDNA and pass one another. It is intended to provide a qualitative overall view to illustrate that the geometry and molecular scale are reasonable and is not intended to illustrate precise atomic details. The process begins with a double-hexamer encircling idealized B-form dsDNA that engages the AAA+ domain DNA-binding hairpins to one strand with a configuration and polarity shown in Fig 1. Concurrently, the N-terminal domain engages its MSSB to the opposing strand with a configuration and polarity shown previously[10] (See Fig. 7). In this arrangement, translocation of ssDNA at one hexamer pulls on the MSSB of the opposing hexamer, twisting the two hexamers with respect to each other about a symmetric interface (the inter-hexamer interface of the orange subunits). The twist aligns two equivalent intra-hexamer subunit interfaces to face each other (the interfaces of the yellow and orange subunits), allowing each hexamer to pull its translocation strand outside of the opposing ring. Several rounds of ATP hydrolysis unwind the strands at the center while concurrently re-winding them beyond the AAA+ hairpins, generating zero net unwinding. The winding of the translocation strand around the complementary strand is similar to winding a spring, which unsprings to exit the interface when the specific ATPase site is in the empty configuration (exits the orange/yellow interface when the orange hairpin is at the top of the staircase and the yellow hairpin is at the bottom; see Fig. 4). Each frame of the left portion was generated with PyMol[5] with subunits colored as in Fig. 2 in cartoon representation. The DNA strands are in cyan and magenta spheres with every 12th nucleotide in white to help show strand motion. Each frame of the right portion was generated with Bobscript[2] in postscript format. Each h2i is shown as a coil and colored as in Fig. 2. The DNA strands are represented as beads on string with varying shades of cyan and magenta. An outline representation for the protein was generated by processing each postscript file with Adobe Illustrator. The frame of reference is looking down the colinear dyad axes of the two hexamers and the two DNA strands.

File Name: Supplementary Movie 8
Description: Factor recruitment consistency with unwinding timing and steric properties of MCM:DNA (0:40; 15 MB; 1684 X 1080).

A movie of Mcm2-7 analogous to Supplementary Movie 7 was generated based on alignments of structures of *Sc*Mcm2-7 with the models of Supplementary Movie 7. The process begins with a slide of the pre-activation state illustrated previously by cryo-EM[15] to a form analogous to the start of Supplementary Movie 7 with the ps1β bound to the leading strand. Each subsequent model consists of an unmodified N-terminal tier from the EM structure of the *Sc*Mcm2-7 double-hexamer (PDB 3JA8[14]) with AAA+ domains from the same structure aligned to the AAA+ positions of the coordinate files used for Supplementary Movie 7. The subunits are uniquely colored with Mcm2 purple, Mcm6 blue, Mcm4 green, Mcm7 yellow, Mcm3 orange, and Mcm5 red. The DNA strands are in cyan and magenta spheres with every 12th nucleotide in white to help show strand motion. The corresponding cumulative degree of dsDNA unwinding is illustrated at the right, calculated as in Fig. 6 to illustrate that the process involves two discrete steps of unwinding consistent with the staged unwinding upon recruitment of Cdc45, GINS, and Mcm10[17]. The structure of Cdc45 (beige) is illustrated to bind at Mcm5/2 according to the previously identified interaction[18] with a timing that has no net unwinding of DNA. The structure of GINS (pink, white, light green, and rose) is illustrated to bind adjacent to Cdc45 according to the previously identified interaction[18] preceding the hinge rotation that generates a small degree of unwinding. Mcm10 binding is expected to precede a large burst of unwinding and the final lagging strand exit from each hexamer, but it is not illustrated in the movie because its structural interface with CMG is not available. Similar to Supplementary Movie 7, the movie is intended to provide a qualitative overall view to illustrate that the geometry and molecular scale are reasonable and is not intended to illustrate precise atomic details. In particular, recruitment of Cdc45 and GINS do not structurally impede any of the protein or DNA transformations that we suggest. The frame of reference is identical to Supplementary Movie 7, looking down the colinear dyad axes of the two hexamers and the two DNA strands. Based on the criteria used to set the initial model, the hinge rotation is predicted be about the interhexamer interface of the two Mcm3 subunits (orange), and the excluded strand is then predicted to exit the interface between Mcm3 and Mcm7 (orange and yellow; see Supplementary Movie 5).
