# Supplementary material for: DNA translocation mechanism of the MCM complex and implications for replication initiation
Source: Nat Commun. 2019 Jul 15;10:3117. doi: 10.1038/s41467-019-11074-3 (PMC6629641; doi:10.1038/s41467-019-11074-3)
Supplement: Supplementary file 12 — Reporting Summary [file 41467_2019_11074_MOESM12_ESM.pdf]

nature research

Corresponding author(s): Eric J. Enemark

Last updated by author(s): Jun 10, 2019

Reporting Summary

Nature Research wishes to improve the reproducibility of the work that we publish. This form provides structure for consistency and transparency in reporting. For further information on Nature Research policies, see Authors & Referees and the Editorial Policy Checklist.

Statistics

For all statistical analyses, confirm that the following items are present in the figure legend, table legend, main text, or Methods section.

n/a	Confirmed	
☐	☒	The exact sample size (*n*) for each experimental group/condition, given as a discrete number and unit of measurement
☐	☒	A statement on whether measurements were taken from distinct samples or whether the same sample was measured repeatedly
☒	☐	The statistical test(s) used AND whether they are one- or two-sided<br>*Only common tests should be described solely by name; describe more complex techniques in the Methods section.*
☒	☐	A description of all covariates tested
☒	☐	A description of any assumptions or corrections, such as tests of normality and adjustment for multiple comparisons
☐	☒	A full description of the statistical parameters including central tendency (e.g. means) or other basic estimates (e.g. regression coefficient) AND variation (e.g. standard deviation) or associated estimates of uncertainty (e.g. confidence intervals)
☒	☐	For null hypothesis testing, the test statistic (e.g. *F*, *t*, *r*) with confidence intervals, effect sizes, degrees of freedom and *P* value noted<br>*Give P values as exact values whenever suitable.*
☒	☐	For Bayesian analysis, information on the choice of priors and Markov chain Monte Carlo settings
☒	☐	For hierarchical and complex designs, identification of the appropriate level for tests and full reporting of outcomes
☒	☐	Estimates of effect sizes (e.g. Cohen's *d*, Pearson's *r*), indicating how they were calculated

Our web collection on statistics for biologists contains articles on many of the points above.

Software and code

Policy information about availability of computer code

Data collection	Diffraction data were collected at SER-CAT beamline 22-ID which uses the program SERGUI to drive the instrument. The 22-ID detector recorded each raw diffraction image using detector-specific software. Diffraction intensities were integrated with the program HKL-2000, which is a very common X-ray data processing package that is available at every beamline we have used.
Data analysis	Data reduction statistics (as reported in Table 1) were derived from the HKL-2000 processing and scaling of the data (see above). Crystallographic refinement was with Phenix 1.12.

For manuscripts utilizing custom algorithms or software that are central to the research but not yet described in published literature, software must be made available to editors/reviewers. We strongly encourage code deposition in a community repository (e.g. GitHub). See the Nature Research guidelines for submitting code & software for further information.

Data

Policy information about availability of data

All manuscripts must include a data availability statement. This statement should provide the following information, where applicable:

- Accession codes, unique identifiers, or web links for publicly available datasets
- A list of figures that have associated raw data
- A description of any restrictions on data availability

The crystallographic coordinates have been deposited at the PDB (accession code 6MII [http://dx.doi.org/10.2210/pdb6MII/pdb]). All data is available from the authors upon reasonable request.

Field-specific reporting

Please select the one below that is the best fit for your research. If you are not sure, read the appropriate sections before making your selection.

☒ Life sciences ☐ Behavioural & social sciences ☐ Ecological, evolutionary & environmental sciences

For a reference copy of the document with all sections, see nature.com/documents/nr-reporting-summary-flat.pdf

Life sciences study design

All studies must disclose on these points even when the disclosure is negative.

Sample size	For diffraction data, the number of total images collected were selected to optimize total data completeness with an X-ray exposure rate that maximized the intensities of individual observations but also was small enough to avoid detrimental radiation decay. The strategy that optimized these factors was determined empirically from test crystals.
Data exclusions	For diffraction data, a single round of "rejections" was applied by the HKL-2000 software in scaling and merging the data. The criteria employed were according to the software's default parameters. We feel this is standard practice, and only a very small fraction of X-ray reflections are rejected. Rejections often correspond to diffraction spots that are partially obscured by detector design or the beamstop.
Replication	For diffraction data, the overall redundancy value corresponds to replicate measurements. For the presented structure, the overall redundancy is 11.1 (Table 1).
Randomization	Diffraction data are collected with a contiguous, sequential sweep of oscillation. This is not random, but the design is somewhat inherent in collecting the most accurate diffraction data. A contiguous sweep (as opposed to a randomized image order) measures diffraction spots that occur over multiple images (for example with half of the intensity measured on one image and the second half on another) directly following one another. This is more accurate for determining the total intensity because the partial measurements all occur with similar levels of radiation decay and do not involve significant re-orienting of the crystal, thus reducing the impact of minor crystal positioning errors. Lastly, the overall start position for the sequential sweep was chosen as the orientation that HKL-2000 calculated to provide full data completeness with the smallest overall oscillation range. This also was chosen to provide the most accurate data by minimizing the effects of radiation decay while achieving the overriding concern of maximizing data completeness.
Blinding	In a sense, we think that the difference electron density maps provided in our manuscript that were generated prior to inclusion of ssDNA or BeF_3 in the model are "blind" to these attributes. These are not affected by model bias for these specific attributes.

Reporting for specific materials, systems and methods

We require information from authors about some types of materials, experimental systems and methods used in many studies. Here, indicate whether each material, system or method listed is relevant to your study. If you are not sure if a list item applies to your research, read the appropriate section before selecting a response.

Materials & experimental systems

n/a	Involved in the study
☒	☐ Antibodies
☒	☐ Eukaryotic cell lines
☒	☐ Palaeontology
☒	☐ Animals and other organisms
☒	☐ Human research participants
☒	☐ Clinical data

Methods

n/a	Involved in the study
☒	☐ ChIP-seq
☒	☐ Flow cytometry
☒	☐ MRI-based neuroimaging
